# Training and Assessment in Urology: Perspective From a Third World Country

**DOI:** 10.7759/cureus.25341

**Published:** 2022-05-25

**Authors:** Rehan N Khan, Amber S Sultan, Nadeem Siddiqui

**Affiliations:** 1 Urology, Aga Khan University Hospital, Karachi, PAK; 2 Surgery, Aga Khan University Hospital, Karachi, PAK

**Keywords:** pakistan, gmc, cpsp, urology training, formative assessment

## Abstract

Formative assessments are an essential yet often overlooked aspect of postgraduate surgical training. This report explores the strategies by which formative assessments are integrated into postgraduate surgical training in Pakistan (using urology as an example), by comparing the regional recommendations and infrastructures offered by local governing bodies to that of a more structured system, as offered by the General Medical Council (GMC). The College of Physicians and Surgeons Pakistan (CPSP) serves as the de facto local accrediting body for postgraduate surgical training and makes a conscious effort in maintaining the standard of training throughout the country. However, although formative assessment activities are encouraged in its roster, they are rarely monitored as strictly as summative outcomes. This is a far cry from how the structured format is exemplified by the GMC’s various guidelines and protocols. It must be emphasized that in order to improve the overall quality of training, measures need to be made to improve the ways in which feedback and formative activities are implemented and monitored.

## Introduction and background

Conventional surgical training has come a long way from the Halstedian model of the early 20th century. Training no longer depends upon the shadowing of “a master of the trade” and following a Byzantine path from “apprenticeship” to “journeyman” to eventually one day become a master oneself. In fact, the classical “see one and do one” format of training has been losing credibility and favor in this day and age of more evidence-based and competency-based education. Supervision must now go hand in hand with structured feedback, repetition, self-reflection, and, in turn, assessment. That being said, both summative and formative modes of assessment are equally important in surgical training, as each plays its own specified role in learning. Although summative assessments focus more on assessing the learner’s knowledge base and aid to monitor one’s performance at the end of a given learning period, the focus of formative assessments is more on encouraging the intrinsic learning motivations of the learner and aiding mentors in tracking the progress of the learner over the span of the learning period.

With regard to the current trend in postgraduate medical education in Pakistan, the quality of training programs is often measured by how well trainees perform during their assessments in exit examinations. Most of the emphasis during surgical training is given to areas that are crucial for their summative assessment at the end of the training [1].

Although continuous formative assessment is an essential component of quality surgical training, considering a lack of formal structures for such assessments and academic trends, it is often hard for institutions to conduct and/or monitor such activities and is thus often neglected and not given its due importance [2].

This paper aims to evaluate the current status and governance of formative assessment during surgical training in our country and compare this to the systems for formative assessments in developed countries, in particular the General Medical Council (GMC).

## Review

The College of Physicians and Surgeons Pakistan (CPSP) as the governing body

The curricula of the various postgraduate training programs offered by the surgical training programs are principally tailored to the needs and requirements of the local accrediting body, the College of Physicians and Surgeons Pakistan (CPSP). According to their website, the CPSP is the premier postgraduate medical training institution of the country, with a focus on improving and accrediting specialist training through standardization of medical practice and training, as well as the promotion of scholarly academics [3]. The CPSP works by promoting clinical practices in various specialties. This aim can be secured by improving the quality of teaching and training, ensuring standardized postgraduate specialty training, conducting summative assessments for awarding college diplomas, and promoting academic research practices.

In order for an institution to be approved by the College, they must adhere to such criteria, after which a formal visitation of the campus by college officials is conducted. However, one major drawback is that although the CPSP does make efforts to ensure poor training institutions are not inducted, there is no formal audit or review conducted by the governing body to keep tabs on such hospitals. The College offers and conducts periodic mandatory workshops, but those are mainly focused on specific areas such as communication skills, technical skills, and research methodology.

The College has set the guidelines and the benchmark standards and monitors and ensures standardization of training among various affiliated institutions in Pakistan. It also ensures that the assessment tools used for assessing the competencies of trainees must be valid and reliable, and the process should be transparent and fair [4].

Formative assessment in urology training

To grasp how candidate assessments are encouraged by the CPSP, a detailed review of the urology handbook was done to see how the assessment has been carried out in this specialty. Interestingly, throughout this entire document, the word “formative” is used only twice, first referring to the quality of feedback the assigned supervisor is expected to give and second to the electronic portfolio, which the candidates are required to keep up to date.

The CPSP is not much involved in how each individual training institution conducts its programs. However, a clear outline is provided of the requirements for recruitment and inclusion into the College for each respective center to follow. Supervisors are all required to have at least five years of independent practice in their respective specialty (after qualifying for fellowship or an equivalent/recognized degree), as well as having partaken in mandatory supervisor training workshops as conducted by the College itself. The College does not however enforce a formal degree in undergraduate or postgraduate education. Feedback is encouraged to be structured and aimed toward being formative for the candidate’s progress. The trainer is required to oversee the candidate’s progress and assist in the learning process. However, no guideline or set requirement of tasks or in-center assessments is suggested. Supervisors are not provided any guidelines on structured training and formative assessment of trainees’ performance [1].

For the specialty of urology, after clearing intermediate modules and doing their specialty training for 3-4 years, they are required to complete certain defined scholarly tasks, as well as regularly update their electronic procedure log [5,6]. This log is essentially a part of the summative exit assessment; however, it is not mentioned how this formative assessment along with feedback is given, despite being labeled as such.

Although the CPSP considers itself to follow a competency-based curriculum, has defined different competencies required for each level of trainees (observer, assistant, performed under supervision, and performed independently), and has made a recommendation that candidate competence should be structured by their level of training, there is no recommendation on how these candidates should be assessed through their training, as its essential focus is to certify that a candidate satisfies the minimum criteria to be a safe practitioner. However, standardization of training has been one of the key objectives of the institution. They in turn play a large impact on the quality of training between various centers, not only in different provinces but also within individual cities.

The GMC as the governing body

In contrast to the CPSP, which focuses primarily on postgraduate training, the GMC covers a multitude of functions relating to clinical practice. It is a public body responsible for maintaining a register of clinical practitioners working in the UK, as well serving as an accrediting body to set the rules and regulations of practice in the country. Among its various roles, the GMC oversees undergraduate medical education and postgraduate specialist training, in addition to the setting of standards of clinical practice and ensuring patient care and safety-related matters.

As evident in their “Guidance on Assessments in Undergraduate Medical Education,” medical schools are encouraged to design their curricula and make appropriate provisions for both summative and formative assessments in their courses [7]. This document explores the benefits of introducing formative assessments to learners, to test for their knowledge, rather than testing “of” learning. Such interactions are suggested to be frequent, informal, and dynamic, and timely, constructive feedback should be offered, which is nonjudgmental toward the learner and focuses primarily on the gaps identified during assessing the competencies of trainees.

The GMC makes sure to carry this approach toward assessment over into postgraduate training, and professional practice, as is described in “Excellence by design: standards for postgraduate curricula guidance on implementation for colleges and faculties” [8]. Here, an emphasis is placed on regular assessments, including constructive formative feedback exercises to be included in the postgraduate curriculum. Assessments must be regular and predefined with the objectives and goals of training kept in consideration. Assessments should cover nine domains, including professional knowledge, professional skills, and professional values and behaviors, which includes health promotion and illness prevention, safety, and quality improvement, as well as education, training, and scholarship, among others. It is suggested that supervisors should try to assess their trainees frequently and sign off areas of professional capability to thus reduce the burden of assessments in the workplace for trainees to progress satisfactorily.

According to their guidance on “Designing and maintaining postgraduate assessment programs,” formative assessments should require and enable interaction between learners, assessors, teams, and patients [9]. Feedback can thus provide insight into one or more areas of performance and can assist in identifying issues of engagement, professional development, and areas of underperformance. Furthermore, input from numerous formative assessments can be monitored, collated, and reviewed periodically.

The GMC describes formative assessment as “Supervised Learning Events” (SLE) and summative assessment as “Assessments of Performance” (AoP) [10]. In order for SLEs to work, trainees and supervisors are encouraged to work together to identify learner-directed learning goals [7]. Such activities have been found to provide an opportunity for the two parties to interact more productively and enhance/promote deeper learning through a combination of regular self-reflection, self-evaluation, and effective feedback. Trainees are encouraged to actively seek out feedback from their faculty and discuss their progress/performance with their supervisors and for them to mutually set goals. Feedback should include developmental outcomes, as decided by both the candidate and their facilitator.

The GMC advocates assessment in the workplace. Thus, an effective tool for Supervised Learning Events is the integration of workplace-based assessments (WPBAs). The GMC emphasizes setting regular SLEs for given competencies and defining a minimum number of relevant assessments, thus following the candidate’s progress through training. Such exercises may also aid in the communication between trainer and trainee. This is particularly important, considering that recent literature elaborates that it is often either ineffective or alternatively, an utter lack of feedback in such activities [7]. Furthermore, trainees may be cautious about seeking feedback from their supervisors, which in turn may reduce the overall academic utility of the assessment. It is thus encouraged for both the trainee and trainer to ensure that every such interaction is accompanied by structured, constructive feedback.

In terms of assessment strategies and functionality, the GMC is very robust and well structured and offers guides and recommendations at multiple levels of training and practice. The GMC emphasizes the utility of formative assessments in its various training protocols and provides a sound structure in its design. Workplace-based assessments are also encouraged as formative tools in clinical training. However, reviewing the CPSP recommendation, workplace-based assessments are suggested, although they are neither encouraged nor advised.

CPSP versus GMC

From a more practical standpoint, it is probably unjust to compare the CPSP to the GMC. Structurally, the GMC is probably more akin to the Pakistan Medical Council (PMC), which is functionally more concerned with the regulation of undergraduate institutions and the registrations of graduate physicians and does not actively involve itself with postgraduate training. This role of postgraduate training thus falls under the domain of the College of Physicians and Surgeons Pakistan alone. This introduces us to another key difference between the two bodies (i.e., GMC and CPSP) in terms of their functionalities. The CPSP, being a validating body, focuses its concern more on the time allocated for the training of the candidates in their respective specialties but does not keep a strict eye on how these candidates will perform once they enter specialty practice. In other words, although validation is a key objective, the College does not emphasize factors such as revalidation or safety in practice.

Taking a glance at institutional practices in Pakistan, it should be emphasized that the programs have been structured to reflect the requirements of the CPSP in general. In some hospitals, the curriculum encourages workplace-based assessments, but these are not routinely monitored by either the individual departments (sections), not by the governing Postgraduate Medical Education (PGME) board. Formative assessments, although advised, are often assumed to have taken place at the end of individually supervised activities and end-of-rotation evaluations; however, there is no written evidence or set criteria suggested on how to conduct such evaluation settings or even how many to conduct for that matter, although, as discussed, this is essentially not required by the overseeing CPSP in the first place.

## Conclusions

One may argue that the end-of-year and in-service examinations conducted by some training institutions, although summative for that certain institution, may serve an overall formative role in the training of the candidate in general. Detailed feedback on trainees’ performance is required, and it should be incorporated to identify the gaps in their learning. Formative assessment should be conducted on a regular basis in every training institution. Trainees can be given detailed feedback on their performance in these institutional assessments and are informed of areas where potential weaknesses are identified. Thus, performances in institution-held assessments may be considered formative exercises for the candidate, preparing them for the eventual fellowship examinations.
